# Highlighting the need for de-implementation – Choosing Wisely recommendations based on clinical practice guidelines

**DOI:** 10.1186/s12913-019-4460-z

**Published:** 2019-09-05

**Authors:** Raija Sipilä, Marjukka Mäkelä, Jorma Komulainen

**Affiliations:** 1 Current Care Guidelines, The Finnish Medical Society Duodecim, Helsinki, Finland; 2 Department of General Practice, University of Copenhagen, Copenhagen, Denmark

**Keywords:** Clinical decision-making, Medical overuse, Guidelines as topic, Choosing wisely

## Abstract

**Background:**

The Choosing Wisely campaign has spread to many countries. Methods for developing recommendations are inconsistent. We describe our process of developing such recommendations from a comprehensive national set of clinical practice guidelines (Current Care, CC) and the results of a one-year Choosing Wisely Finland project.

**Methods:**

Two of the authors drafted the quality and process criteria for all the Choosing Wisely Finland recommendations. The quality criteria were relevance, feasibility, evidence-based and strength. These were discussed in editors’ meetings and subsequently revised. Two different processes for developing recommendations within national clinical practice guidelines were designed and piloted (processes A and B). Process A was based on a published guideline. The recommendations are drafted by an editor and revised and approved by the guideline development group. In process B the development of the recommendations is integrated with guideline production or update. Choosing Wisely recommendations were then drafted for half of the published CC Guidelines. An additional process (process C) was designed for producing independent recommendations outside a guideline.

**Results:**

At least one Choosing Wisely recommendation could be identified from 39 out of 52 reviewed guidelines. Of the 106 recommendations drafted, 62 (58%) were accepted for publication. The main reasons for rejection were inability to give a strong recommendation (*n* = 18, 41%) and insufficient relevance (*n* = 14, 32%). Two thirds (*n* = 41, 66%) of the published recommendations were based on high to moderate level of evidence, and 18% (*n* = 11) on low or very low level of evidence, whereas for the rest, the quality of evidence was not critically appraised.

**Conclusions:**

Choosing Wisely recommendations can be produced systematically from existing clinical practice guidelines. The rigorous methods of evidence-based medicine ensure high-quality recommendations. We welcome the use of our processes and methods describes in this article by other guideline-producing organizations.

**Electronic supplementary material:**

The online version of this article (10.1186/s12913-019-4460-z) contains supplementary material, which is available to authorized users.

## Background

De-implementation means reducing or stopping the use of a health service or practice provided to a patient. Reduction is appropriate in cases where interventions are used inappropriately or have low value, e.g. interventions that are shown to be ineffective or harmful [1]. Low-value interventions can have an unfavorable balance between harms and benefits or be more costly than optional interventions. De-implementation should also be considered for interventions for which the evidence is uncertain or lacking.

Identifying and prioritizing interventions that should be avoided is a key challenge [2]. The Choosing Wisely campaign was launched in 2012 to support discussions between physicians and patients [3] about avoiding low-value care. The campaign invited medical specialty societies in the US to produce top 5 lists of low-value services. Each society was free to develop lists with their own methods using documented, publicly available processes. The criteria for topic selection were that the procedure is used frequently, carries a significant cost, may expose patients to harm or burden, or may increase strain on the health care system. Each recommendation should be supported by strong evidence [1]. The campaign has spread to over 20 countries [4]. In Germany a manual and criteria have been published for developing Choosing Wisely recommendations [5].

In Germany, several methodological challenges in producing Choosing Wisely recommendations were identified [6]. These include weak methodology, lack of transparency about prioritization of topics and unrealistic expectations for the recommendations as a solution to overuse. As one solution to these challenges, using high-quality clinical practice guidelines as a starting point was suggested. This would secure systematic literature searching and appraisal, multidisciplinarity, and consensus with independent moderation. In addition, the viewpoint would change from medical specialties to diseases.

In Finland, there is a library of 105 national evidence-based Current Care (CC) Guidelines. Here we describe methods for developing the Choosing Wisely Finland recommendations from these guidelines, and for integrating such recommendations back into the guidelines.

## Methods

The work was inspired by international experiences, including Choosing Wisely campaigns as well as NICE’s Do-not-do -recommendations. We developed a methodology based on the scanty literature available, piloting methods on the way. We worked in close collaboration with guideline editors, a group of physicians with training in critical appraisal and facilitating guideline development groups.

### Setting

The Finnish Medical Society Duodecim, in co-operation with medical specialty societies, has produced national clinical practice guidelines (Current Care) since 1994 [7]. The guidelines cover prevention, diagnostics, medical treatment, and rehabilitation of diseases. CC Guidelines serve as a support for treatment decisions for healthcare professionals, with a particular emphasis on primary care. They are available for anyone through open access, and most of them include a patient version. CC Guidelines are accessed over 6 million times a year. Evidence based health care has a long history in Finland. In addition to guidelines, systematically produced health technology assessments (HTAs) have guided the implementation of new technologies in hospitals, including consensus recommendations to limit the spread of low-value technologies [8]. The Council for Choices in Health Care in Finland produces recommendations and defines the service basket at a societal level [9].

The production of CC Guidelines follows international standards of development [10]. The level of evidence is graded from A to D (high to very low) [11] based on the system launched by the Institute of Medicine, as endorsed by the Guidelines International Network [12]. Guideline production is publicly funded and led by the medical profession. The editorial team includes 11 editors representing different medical specialties. They act as project managers and evidence-based medicine (EBM) methodologists. All guideline development groups are multiprofessional.

The Finnish health care system is decentralized, organized by municipalities and funded by taxation, apart from occupational health care which is funded by employers and produced largely by private companies. Specialized health care is provided in 20 hospital districts, five of which have a university hospital. CC Guidelines are well-known and widely accepted by clinicians and used also at organizational level [13], as all Finnish hospital districts expect their entire staff to apply CC Guidelines in care pathways.

### Process

We decided to base our de-implementation work on the existing comprehensive national guideline library. The Ministry of Social Affairs and Health funded the pilot project. The first step was to set up criteria for the recommendations. Although Choosing Wisely methods are described by the participating societies on the Choosing Wisely website, there were few scientific publications on the methodology. For Choosing Wisely Finland, we aimed to describe criteria that guaranteed transparent, consistent, and EBM-based methodology and that was suited for guideline producers. In line with the US Choosing Wisely criteria relevance for patients and health care providers was considered important. To limit the number of recommendations, feasibility was added as a criterion. Transparency was primary consideration when defining process criteria. The criteria were drafted by the CC Editor-in-Chief (JK) and Managing Editor (RS) and discussed in editors’ meetings. As a result, process criteria were separated from quality criteria for the recommendations (Table 1).

**Table 1:** Criteria for the Choosing Wisely recommendations

Quality criteria	
Relevance: The recommendation should target an intervention that • can cause significant harm to patients or the health care system or • is ineffective but widely used or • has a notable economic impact	
Feasible: Possibility to have an impact on clinical practice	
Evidence-based: The recommendation is based on critically appraised literature, preferably presented in evidence summaries	
Strength: A possibility to give a strong recommendation* (GRADE criterion) [14]	
Process criteria	
The reasons for acceptance or rejection are documented	
The authors of the recommendation do not have significant conflicts of interest	
The recommendations are published in a consistent format including a short justification	
The Finnish Medical Society has the final decision on publishing	

*A strong recommendation can in certain cases be given independent of the level of evidence

We developed a structured format for publishing [See Additional file 1]. According to the principles of EBM, each recommendation is accompanied by a justification, references and, when appropriate, an evidence summary. A short list of possible barriers for implementation is provided when relevant.

We identified three different processes for developing Choosing Wisely Finland recommendations (Table 2) and discussed these with the editors. Two of these are based on evidence gathered in a guideline production process. In process A, the Choosing Wisely recommendations were drawn from and incorporated into an existing guideline. In process B, the development of recommendations was integrated to the guideline development process. The third process (Table 2, process C) can be used when a national guideline on the subject is not available.

**Table 2:** The processes for developing Choosing Wisely recommendations in Finland

	Process A. Based on a published guideline	Process B. Integrated with guideline production or update	Process C. Independent recommendation
Step 1. Topic proposal	An editor reviews the guideline for possible recommendations	A proposal from a guideline development group member, discussed in the group	A proposal from a medical specialty society, other organization or individual
Step 2. Recommendation draft with justification	The editor based on the guideline and evidence summaries	Group member based on literature search and critical appraisal	An editor based on literature search and critical appraisal
Step 3. Review and revision	Revisions based on comments from the chair and relevant guideline development group members. Final approval by the Editor-in-Chief (documented). Informing the group and possible comments.	Comments from the group. External review (of the guideline draft). Possible revisions. Final approval by the Editor-in-Chief (documented).	Comments from 1 to 2 topic experts, possible revisions. External review (of the recommendation): e-questionnaire, quality criteria assessed with a Likert scale from 1 to 5. Possible revisions. Final approval by the Editor-in-Chief (documented).
Step 4. Publishing	Incorporating the recommendations into the guideline. Technical editing and publishing.	Incorporating the recommendations into the guideline. Technical editing and publishing.	Technical editing and publishing.

In the pilot phase, five CC Guidelines were reviewed according to process A (Table 2) and eight Choosing Wisely recommendations were drafted. The Ministry of Social Affairs and Health then funded a project for developing relevant Choosing Wisely recommendations from 50 existing CC Guidelines. In cooperation with the Ministry, a set of recently updated guidelines were selected from the national guideline library.

The editors were initially introduced to the Choosing Wisely ideology in a pilot phase workshop. Subsequently, the editors were trained to understand low-value care, and learn the processes and quality requirements of Choosing Wisely Finland recommendations. The training included educational sessions, presentations as well as feedback and problem solving.

Each editor received a set of guidelines to review. The editors were asked to draft up to 5 recommendations per each guideline. They identified topics and drafted recommendations using the existing guidelines and their evidence summaries as a basis (Table 2, process A) and proceeded with the most relevant ones. The recommendations could target treatment, diagnostic testing, screening, rehabilitation or follow-up. Topics were often identified and drafted in close co-operation with the chair of the guideline development group and members responsible for the topic. Recommendations were discussed at subsequent editors’ meetings. Before final approval, the guideline development group members were asked to comment on the recommendations and consider relevance and other criteria. Consensus was reached by discussions. The Managing Editor led the project, tutored other editors and provided detailed individual feedback.

## Results

Altogether 52 guidelines were reviewed during the project. In 13 (25%) guidelines, no recommendations fulfilling the Choosing Wisely criteria were identified. For the remaining 39 guidelines, 106 recommendations were drafted, and 62 (58%) of these were accepted for publication [see Additional file 2]. Because relevance was considered to be insufficient, recommendations were rejected in 14 cases. In another 8 cases the underlying evidence was judged to be too weak for a strong recommendation according to GRADE criteria. In 18 cases a strong recommendation against an intervention was not possible due to the complexity of the issue in the Finnish health care setting. In four cases the evidence was outdated, and a new literature search and review would have been required.

A third of the published recommendations were related to pharmacotherapy (*n* = 21), whereas a quarter dealt with diagnostics (*n* = 15). None were related to psychosocial treatments or rehabilitation. Twenty-two of the 62 recommendations were supported by high-level of evidence, 19 by moderate level, 10 by low level and 1 by very low level of evidence. In 10 cases an evidence summary was considered unnecessary, typically because they dealt with overdiagnosis, overtreatment or well-known, and potentially serious adverse events. A typical justification for a Choosing Wisely recommendation was an unfavorable balance between benefits and harms or ineffectiveness of the intervention (Table 3).

**Table 3:** Justification categories for Choosing Wisely recommendations with examples. The categories are modified from Choosing Wisely principles and the GRADE criteria

Category	Number of recommendations*	Guideline: Choosing Wisely recommendation	Justification (level of evidence)
Unfavorable balance between benefits and harms	27	Urinary tract infection: Do not treat asymptomatic bacteriuria in the elderly, because it does not decrease incontinence, urinary tract infections or mortality.	Eradication of bacteriuria does not decrease incontinence, urinary tract infections or mortality. Antibiotics have side effects and increase antimicrobial resistance. (A)
Ineffective treatment or insensitive diagnostic test	22	Tendon disorders of the shoulder: Do not use ultrasound to treat tendon disorders of the shoulder.	Ultrasound therapy is not more effective than placebo (pain, function). (A)
Unnecessary intervention	11	Stable Coronary Artery Disease: Do not do an exercise test for a patient with chest pain and low probability of coronary artery disease.	In this patient group the likelihood of a false positive result is high. (no evidence summary) [22, 23]
Alternative treatment options more effective	8	Acute otitis media: Do not routinely treat tympanostomy tube otorrhea with oral antibiotics.	Topical antibiotics are more effective than oral antibiotics in the treatment of tympanostomy tube otorrhea. Oral antibiotics have more side effects and increase antimicrobial resistance. (B)
Unfavorable balance between benefits and costs	3	Age-related macular degeneration: Avoid ranibizumab and aflibercept as first line treatments for age related macular degeneration due to high costs.	Comparable effectiveness but higher costs than other treatment options. (B)
No evidence for effectiveness	2	Glaucoma: Do not routinely measure diurnal fluctuations in intraocular pressure following monitoring progression of glaucoma.	The benefit is uncertain. There is little research and its quality is low. (D)

*A recommendation could be classified into several categories

## Discussion

In contrast to most Choosing Wisely campaigns that start by identifying isolated" Things Providers and Patients Should Question", we utilized existing clinical practice guidelines. We also developed and applied systematic evidence-based methods to secure the trustworthiness of the recommendations. Methodological requirements in the US Choosing Wisely campaign are rather loose (e.g. being evidence-based) and the reported methodologies vary from Delphi method to adopting other medical societies’ recommendations [1].

A major strength of our work is that the recommendations are developed systematically and transparently, using EBM methodology. According to one report, only 32% of Choosing Wisely recommendations were judged sufficiently trustworthy [14]. Admon et al. extracted all 320 recommendations published by the Society of Hospital Medicine and found that a majority (70%) were referring to guidelines of variable quality while only one in five was linked to primary research [15].

According to GRADE, a strong recommendation should usually be based on at least moderate confidence in effect estimates [16]. Of our Choosing Wisely recommendations, 66% were based on a high to moderate level of evidence. For 16% the quality of the evidence was not formally assessed in the guideline. One study found that under 10% of the US Choosing Wisely recommendations were based on low-quality evidence [15], but in the primary care context, the majority of recommendations were self-evident, e.g. based on “consensus or disease-oriented evidence” [17]. We have not formally compared our Choosing Wisely recommendations to the US recommendations but made some interesting observations. It seems that our criteria result in fewer recommendations per topic or disease. According to our results, the main reasons for rejecting a recommendation were a lack of relevance and complexity of the issue. Some recommendations may be acceptable in one setting but not in another one. For example, for type 2 diabetes, the US library has a recommendation to “Avoid routine multiple daily self-glucose monitoring in adults with stable type 2 diabetes on agents that do not cause hypoglycemia.” [1] We suggested a similar recommendation, but our guideline development group assessed the issue to be too complex as there are some patient groups that may benefit from such monitoring. The guideline development group pointed out that municipalities already have restricted the distribution of test strips.

Our method has several other strengths. The criteria are clearly described, making the process transparent. Each recommendation also includes a justification and, if appropriate, an evidence summary. The justification provides detailed reasoning for the reader. This is especially important if the evidence is of low quality or lacking. The reader thus gets an opportunity to make informed decisions to avoid low-value care and receives tools for shared decision-making. We also argue that the process changes the viewpoint from specialty-oriented to more disease-oriented when a guideline development group formulates recommendations. Furthermore, a multiprofessional group probably evaluates the relevance of a recommendation more widely than a group from one specialist society.

One limitation of our process is that the quality criteria are not formally assessed when producing recommendations from guidelines with processes A and B. The recommendations are, however, based on evidence gathered and assessed by a guideline development group during the guideline process. A more formal assessment would naturally increase the transparency of the process. A more extensive formal review is planned for recommendations that are not based on a guideline (Table 2, process C). Both quality and process criteria are checked by the Editor-in-Chief before final approval. At this point, in particular the level and quality of evidence as well as the possibility to give a strong recommendation are ascertained.

Another limitation is that our guideline development groups do not include patients. We plan to involve lay representatives in guideline production, including the steps for Choosing Wisely Finland recommendations. This may increase the relevance of the recommendations.

Forty-four of the draft recommendations were not published. The main reason for this was that we were unable to give a strong recommendation against an intervention due to complexity of the issue at hand. This underlines the need to discuss evidence and reach a consensus to avoid oversimplification and conflicts. We did not use a full GRADE process, where the importance of each outcome is weighed and tabulated. This would likely be useful when guidelines are updated and new Choosing Wisely recommendations are drafted.

Conflict of interest declarations are gathered from all authors during CC Guideline production or updates (Process B, Table 2). However, for the other two processes, formal updated declarations are not requested as of yet.

Awareness is the first step to change. To raise awareness of our recommendations we use our website, social media, press releases and sessions at continuing medical education events. Selected recommendations are also published in the scientific journal of the Finnish Medical Society Duodecim. Choosing Wisely campaigns aim to facilitate discussions between physicians and patients. The US campaign includes posters and videos for the public. Relevant Choosing Wisely recommendations are incorporated into the patient versions of CC Guidelines. To produce more extensive material to the public, however, we would need more resources and co-operation with patient organizations. So far, we have not studied changes in awareness or the actual impact of our recommendations. In the US, change in awareness was negligible during the campaign in 2014–2017 [18]. Some small changes in intervention prevalence have been detected, but the clinical impact of the decrease has remained questionable [19].

Guideline implementation research shows that dissemination is not enough to change practices; active interventions are needed. There is little research on de-implementation and most behavioral theories do not differentiate between trying to increase and decrease the frequency of a behavior [20]. Typical major barriers to avoiding low-value care include malpractice concerns, time pressures in the clinical encounter, patient demand, and physicians’ need for more information to reduce uncertainty [18, 21]. Choosing Wisely recommendations are one tool to facilitate change, but other interventions would be needed to overcome barriers.

## Conclusions

We have shown that it is feasible to produce Choosing Wisely recommendations systematically from existing evidence-based clinical practice guidelines. A parallel production of guidelines and Choosing Wisely recommendations has obvious benefits: duplicate work is avoided and rigorous EBM methods and processes ensure high quality recommendations. Other guideline producers are welcome to make use of our processes and methods.

## Additional files

Additional file 1: Template and an example for a Choosing Wisely recommendation, Current Care Guidelines. A structured template for publishing Choosing wisely recommendations used in the project and one example of a Choosing Wisely Finland recommendation. (DOCX 19 kb)

Additional file 2: A list of reviewed Current Care guidelines and accepted and rejected Choosing Wisely recommendations. A list of guidelines reviewed during the project, all drafted Choosing Wisely recommendations and reasons for rejection. (DOCX 38 kb)

## Data Availability

All data generated or analyzed during this study are included in this published article and its supplementary information files.

## Abbreviations

CC: Current Care
EBM: Evidence-based medicine
HTA: Health technology assessment
