# Supplementary material for: Highlighting the need for de-implementation – Choosing Wisely recommendations based on clinical practice guidelines
Source: BMC Health Serv Res. 2019 Sep 5;19:638. doi: 10.1186/s12913-019-4460-z (PMC6729023; doi:10.1186/s12913-019-4460-z)
Supplement: Supplementary file 1 — Template and an example for a Choosing Wisely recommendation, Current Care Guidelines. A structured template for publishing Choosing wisely recommendations used in the project and one example of a Choosing Wisely Finland recommendation. (DOCX 19 kb) [file 12913_2019_4460_MOESM1_ESM.docx]

# **Additional file 1.** **Template for Choosing Wisely Finland recommendations, Current Care Guidelines and an example**

Headline (Short description of the subject based on PICO)

Date, Authors

**Choosing wisely recommendation** (Using standard expressions depending on certainty of evidence)

Brief justification for the recommendation in non-technical language.

| References | The Current Care Guideline and other references |
| --- | --- |
| Evidence summary | Link to the evidence summary |
| Additional information | Link to additional information on the subject included in the guideline (if available) |
| Barriers and facilitators for implementation | Short list of possible barriers and facilitators related to health care organizations, professionals, patient and society |

Pap smear testing for young women

Marjukka Mäkelä, Karolina Louvanto, Tanja Laukkala, and Pekka Nieminen

25.5.2018

**Avoid performing untargeted pap smear testing in asymptomatic young women (under 25 years), because the cervical cell abnormalities in most cases regress spontaneously.**

In young women, under age 25, most HPV (human papilloma virus) infections will regress spontaneously. According to two follow-up studies, 62-81% will spontaneously regress in 12 months, and 91% in three years. New infections among sexually active women are common [1]. Repeated testing may, therefore, reveal new infections, caused by different types of viruses, although the spontaneous regress is likely.

| References | The Current Care Guideline: Cytological changes in the cervix, vagina and vulva. Working group set up by the Finnish Medical Society Duodecim, the Finnish Colposcopy Association. Helsinki 2016. www.kaypahoito.fi.  [1] Louvanto K, Rintala MA, Syrjänen KJ, Grénman SE, Syrjänen SM. Incident cervical infections with high- and low-risk human papillomavirus (HPV) infections among mothers in the prospective Finnish Family HPV Study. BMC Infect Dis 2011;11:179 |
| --- | --- |
| Evidence summary | The mean duration of a HPV infection in women aged 13-23 years is eight months, and up to 90% will recovered within 2 years (B) (<https://www.kaypahoito.fi/dnd00077>) |
| Additional information | Not available |
| Barriers and facilitators for implementation | Barriers:  Clinical custom of yearly testing  Other reasons for testing  Lack of knowledge  Facilitators:  Additional costs of testing |
