# Supplementary material for: Highlighting the need for de-implementation – Choosing Wisely recommendations based on clinical practice guidelines
Source: BMC Health Serv Res. 2019 Sep 5;19:638. doi: 10.1186/s12913-019-4460-z (PMC6729023; doi:10.1186/s12913-019-4460-z)
Supplement: Supplementary file 2 — A list of reviewed Current Care guidelines and accepted and rejected Choosing Wisely recommendations. A list of guidelines reviewed during the project, all drafted Choosing Wisely recommendations and reasons for rejection. (DOCX 38 kb) [file 12913_2019_4460_MOESM2_ESM.docx]

# Additional file 2 A list of reviewed Current Care guidelines and accepted and rejected Choosing Wisely recommendations.

| **Guideline (year of publication)** | **Choosing Wisely recommendation or topic of a recomendation** | **Level of evidence or references** | **Decision** | **Reason for rejection** |
| --- | --- | --- | --- | --- |
| ADHD (Attention-deficit hyperactivity disorder) (2017) | Do not recommend polyunsaturated fatty acids for treatment of children and adolescents with ADHD. | B | Accepted |  |
|  | Do not order EEG-neurofeedback for treatment of children and adolescents with ADHD. | B | Accepted |  |
| Asthma (2012) | House dust mite control in treatment of asthma is unnecessary, because it doesn't have effect on asthma symptoms or need for medication. | B | Rejected | Insufficient relevance |
| Atrial fibrillation (2017) | Avoid cardioversion in elderly patients with atrial fibrillation and only mild symptoms. | A | Accepted |  |
| Brain injuries (2017) | Do not order a skull x-ray for patients with suspected brain injury. | [1, 2, 3, 4, 5, 6, 7] | Accepted |  |
|  | Do not use prophylactic hypothermia in treatment of patients with brain injury. | A | Rejected | Insufficient relevance |
| Caries (2014) | Do not advice children and adolescents to use non-fluoridated toothpaste as it has a poor effect on caries control. | A | Accepted |  |
|  | Do not advice children to rinse their mouth vigorously after brushing the teeth, as this apparently weakens the effectiveness of fluoride toothpaste | C | Accepted |  |
| Chronic leg ulcers (2014) | Avoid systemic antibiotics in treatment of venous leg ulcers, because they seem not to shorten the time for healing but do increase the incidence of resistant bacteria. | C | Accepted |  |
|  | Venous surgery and chronic venous ulceration. | B | Rejected | Too weak evidence |
|  | Avoid using long-term topical treatment, because it predisposes to allergic reactions. | B | Rejected | Too weak evidence |
| Coronary event: unstable angina pectoris and cardiac infarction without ST elevation (2014) | Same Choosing wisely recommendation with STEMI guideline. |  | Accepted |  |
| Cytological changes in the cervix, vagina and vulva (2016) | Avoid treating low-grade squamous intraepithelial cervical lesions because these infectious changes mostly regress during a few years' follow-up. | A | Accepted |  |
|  | Avoid performing untargeted pap smear testing in asymptomatic young women (under 25 years), because the cervical cell abnormalities in most cases regress spontaneously. | B | Accepted |  |
| Depression (2016) | Avoid drugs as the first line treatment for a mild or moderate depression in young persons. | A | Accepted |  |
|  | Avoid quetiapine as the only treatment for patients with a major depressive disorder without special reasons and a psychiatric consultation. | A | Accepted |  |
| Distal radius fracture (2016) | Do not repeat reposition of a distal radius fracture, if it dislocates during cast treatment. | D | Accepted |  |
|  | Do not immobilize a distal radius fracture in ulnar flexion but rather in functional position. | C | Rejected | Too weak evidence |
| Dyslipidemias (2017) | Do not start lipid lowering medication without assessing the overall risk of cardiovascular disease. | A | Accepted |  |
|  | Do not prescribe large doses of simvastatin (80 mg/day), since it may increase the risk of muscle pain. | [8, 9, 10] | Accepted |  |
|  | Do not routinely stop statins at 80 years of age. | No evidence summary available | Rejected | Complex issue |
| Eating disorders (2014) | Avoid using contraceptives for restoring menstruation in anorectic patients since they may harm bone density. | B | Accepted |  |
|  | Do not routinely add drug treatment to support psychotherapy in patients with eating disorders. | B | Accepted |  |
|  | Avoid unnecessary inpatient care of patients with eating disorders. | No evidence summary available | Rejected | Complex issue |
|  | Nutrition treatment in patients with eating disorders. | No evidence summary available | Rejected | Too weak evidence |
| Endodontic treatment (2016) | Avoid root canal treatment for treatment of deep caries lesions, and preserve the living tooth by partial or phased caries removal. | B | Accepted |  |
|  | Don’t prolong root canal treatment, try to complete it during 1 to 3 appointments. | No evidence summary available | Rejected | Too weak evidence |
| Epilepsies (adult) (2014) | Do not order routine follow-up blood samples in adult patients treated with anti-epileptic drugs. | No evidence summary available | Rejected | Outdated evidence |
|  | It is unnecessary to use anti-epileptic drugs for preventing seizures following acute traumatic brain injury, because it does not prevent posttraumatic epilepsy. | A | Rejected | Insufficient relevance |
| Epilepsy and febrile seizures (children) (2013) | Avoid antipyretic drugs (paracetamol, ibuprofein, diclofenac) for prevention of febrile seizures in children. | A | Accepted |  |
|  | Prevention of epileptic seizures with anti-epileptic drugs after the first seizure. | B | Rejected | Insufficient relevance |
|  | Do not order routine follow-up blood samples in patients treated with anti-epileptic drugs. | No evidence summary available | Rejected | Outdated evidence |
| Food allergy (children) (2015) | Do not introduce diagnostic testing for food allergies in children with mild atopic eczema. Do not routinely do such testing in infants with moderate atopic eczema. | A | Accepted |  |
|  | Do not order serum food specific IgA or IgG antibody tests for diagnosing food allergies. | [11, 12] | Accepted |  |
|  | In a primary care setting, do not advice a lactating mother to use allergy diet when her baby is suspected of having cow milk allergy or diagnosed with food allergy. | B | Accepted |  |
| Glaucoma (2014) | Do not routinely measure diurnal fluctuations in intraocular pressure in monitoring progression of glaucoma. | D | Accepted |  |
| Heart failure (2017) | Avoid glitazones in diabetic patients with heart failure. | A | Accepted |  |
|  | When treating systolic heart failure, avoid calcium channel blockers with predominantly cardiac effects (diltiazem, verapamil) and short-acting nifedipine. | B | Accepted |  |
|  | Avoid NSAID drugs in patients with heart failure. | A | Accepted |  |
|  | When treating heart failure, avoid adding angiotensin blocker to the combination of angiotensin-converting enzyme inhibitor and mineralocorticoid receptor blocker. | No evidence summary available | Rejected | Insufficient relevance |
| Hip fracture (2017) | Do not restrict motion of persons in inpatient or residential care to decrease the risk of hip fractures. | B | Accepted |  |
|  | Do not use joint endoprosthesis without cement in treating hip fractures. | A | Accepted |  |
| Insomnia (2017) | Avoid prescribing benzodiazepins for insomnia as they have multiple harmful side effects. | A | Accepted |  |
|  | Do not prescribe melatonin for insomnia caused by a primary disease or shift work. | A | Accepted |  |
|  | Avoid sedatives for sleeping disorders in patients aged 60 years or more. | A | Accepted |  |
| Ischemic stroke and TIA (2016) | Avoid treating stroke patients of any age in a general ward if a special unit for cerebrovascular disorders is available. | A | Rejected | Complex issue |
| Knee and hip osteoarthritis (2014) | Avoid glucosamine in patients with arthritis, since its effectiveness does not differ from placebo treatment. | A | Rejected | Complex issue |
|  | Avoid chondroidin sulfate in patients with arthritis, since its effectiveness does not differ from placebo treatment. | A | Rejected | Complex issue |
| Memory diseases (2017) | Do not continue treatment of memory disease with same drug if it does not show an effect during 6 months of follow-up. | No evidence summary available | Accepted |  |
|  | Do not use combination therapy with cholinesterase inhibitors and memantine for patients with mild Alzheimer's disease. | A | Accepted |  |
|  | Avoid long-term use of antipsychotics for dementia patients with mild psychotic behavioural symptoms. | B | Accepted |  |
|  | Ginkgo bilobain treatment of memory diseases. | B | Rejected | Complex issue |
|  | Anticolinergic drugs for patients with memory disease. | No evidence summary available | Rejected | Complex issue |
| Migraine (2015) | Avoid botulinum toxin A in treatment of episodic migraine. | A | Accepted |  |
|  | Use of opioids in treatment of migraine. | No evidence summary available | Rejected | Insufficient relevance |
| Multiple sclerosis (2015) | Avoid using interferon beta for secondary progressive multiple sclerosis since it apparently does not prevent disease progression. | B | Accepted |  |
|  | Do not use glatiramer acetate for secondary progressive multiple sclerosis. | D | Rejected | Outdated evidence |
| Obesity (adults) (2013) | Do not aim for a normal body mass index (BMI<25 kg/m2) in obese (BMI>30 kg/m2) persons. Health care professionals should support the patient in setting realistic goals. | [13] | Accepted |  |
| Osteoporosis (2018) | Do not screen for osteoporosis with bone density measurements from patients who don't have an increased risk of fractures. | No evidence summary available | Accepted |  |
|  | Do not repeat bone density measurement within 2 years of the previous measurement without a special reason. | No evidence summary available | Rejected | Lack of evidence |
|  | Do not recommend bed rest for longer than 2 to 3 days for a patient with a vertebral fracture. | No evidence summary available | Rejected | Insufficient relevance |
| Otitis media (acute) (2017) | Do not routinely treat tympanostomy tube otorrhea with oral antibiotics. | B | Accepted |  |
|  | Do not use broad-spectrum antimicrobial agent as a first line treatment of otitis media in children. | C | Rejected | Complex issue |
|  | Do not treat otitis media with antimicrobials for longer than 7 days without a special reason. | B | Rejected | Insufficient relevance |
|  | Do not routinely schedule a follow-up examination after otitis media. | No evidence summary available | Rejected | Too weak evidence |
| Pain (2017) | Avoid using NSAIDs in patients with cardiovascular disease or its risk factors. | B | Accepted |  |
|  | Avoid using NSAIDs in patients with anticoagulant medication or a risk of gastrointestinal bleeding. | [14 ,15] | Accepted |  |
|  | Avoid combining tramadol or other opioids with tricyclic antidepressants, SSRIs, SNRIs, or moclomebid. | [16, 17] | Accepted |  |
| Periodontal diseases (2016) | Avoid tooth implants in smoking patients with parodontitis, if they do not commit to supportive dental care. | A | Accepted |  |
| Peripheral arterial disease (2010) | Bypass in severe ischaemia of the leg. | C | Rejected | Outdated evidence |
| Pharyngitis (2013) | Do not take a throat swab from patients with mild sore throat symptoms. | A | Accepted |  |
|  | Do not prescribe broad spectrum antibiotics for patients with sore throat. | C | Accepted |  |
| Post-traumatic stress disorder (PTDS) (2014) | Propranolol in prevention of PTDS. | B | Rejected | Insufficient relevance |
|  | Structured trauma-oriented psychotherapy for refugees and asylum seekers | B | Rejected | Insufficient relevance |
|  | Trauma-oriented psychotherapy for PTSD of over three months | B | Rejected | Insufficient relevance |
| Psoriasis (skin and joints) (2017) | The increased risk for squamous cell cancer of the skin in patients treated with PUVA followed by ciclosporin. | No evidence summary available | Rejected | Insufficient relevance |
| Repetitive strain injuries of the hand and forearm (2013) | Do not use local corticosteroid injections in treatment of lateral epicondylitis. | A | Accepted |  |
| Rheumatoid arthritis (2015) | Avoid large corticosteroid doses (over 10 mg prednisolon per day) in treatment of rheumatoid arthritis. | C | Accepted |  |
| Shortened dental arch (SDA) (2013) | Avoid molar teeth extraction and shortening dental arch especially when incisor contacts are missing, the patient has advanced periodontitis, temporomandibular disorders or unstable dental occlusion. | B | Accepted |  |
| Sleep apnoea (obstructive sleep apnoea in adults) (2017) | Do not establish a diagnosis of obstructive sleep apnoea based only on oximetry performed at home. | C | Accepted |  |
|  | Do not treat mild or moderate sleep apnoea with a radiofrequency operation without special reasons. | C | Accepted |  |
|  | Do not treat mild obstructive sleep apnoea with laser assisted operation of uvula palatina. | C | Accepted |  |
| Stable coronary artery disease (2015) | Do not order an exercise test for chest pain patients with a low pretest probability of coronary artery disease. | [18, 19] | Accepted |  |
|  | Avoid ivabradin for patients with stable coronary artery disease who do not have heart failure. | No evidence summary available | Rejected | Insufficient relevance |
|  | Treatment of stable coronary artery disease with betablockers. | B | Rejected | Complex issue |
|  | Do not use computed tomographic angiography for ruling out coronary artery stenosis in individuals with chest pain and high pretest probability of the disease, or for screening asymptomatic patients. | No evidence summary available | Rejected | Complex issue |
| STEMI (2011) | Avoid giving extra oxygen routinely to patients with acute myocardial infarction. | C | Accepted |  |
|  | Do not settle for less than a 14-lead electrocardiogram in diagnosing an acute myocardial infarction. | A | Rejected | Complex issue |
| The tendon disorders of the shoulder (2014) | Do not treat subacromial impingement syndrome by operation. | A | Accepted |  |
|  | Do not use ultrasound to treat tendon disorders of the shoulder. | A | Accepted |  |
| Treatment of alcohol abuse | Avoid prescribing addictive drugs to patients with problematic alcohol use without special reasons and caution. | C | Accepted |  |
|  | Do not drink alcohol during pregnancy. | No evidence summary available | Rejected | Complex issue |
|  | Avoid drinking large amounts of alcohol, if you are elderly. | No evidence summary available | Rejected | Complex issue |
| Type 2 diabetes (2018) | Avoid routine daily self-glucose monitoring in adults with stable type 2 diabetes on drugs that do not cause hypoglycemia. | No evidence summary available | Rejected | Complex issue |
|  | Avoid intensive glucose lowering in patients with long-term type 2 diabetes | A | Rejected | Complex issue |
| Urinary incontinence (women) (2017) | Do not use systemic hormone replacement therapy for urinary incontinence in postmenopausal women. | C | Rejected | Insufficient relevance |
|  | \| Avoid anticholinergic drugs in elderly patients due to side effects. \| \| --- \| | C | Rejected | Too weak evidence |
| Urinary tract infections (2015) | Avoid antiseptic-coated urethral catheters during short-term (under 30 days) catheterisation. | B | Accepted |  |
|  | Do not treat asymptomatic bacteriuria in the elderly, because it does not decrease incontinence, urinary tract infections or mortality. | A | Accepted |  |
|  | Avoid taking urine samples from healthy women aged 18-65years with typical cystitis symptoms and no risk factors. | A | Rejected | Complex issue |
| Venous insufficiency of the lower limb (2016) | Do not routinely use compression therapy for patients with symptomatic uncomplicated venous reflux disease. | B | Accepted |  |
|  | Do not use other imaging than ultrasound for diagnosing venous reflux disease. | No evidence summary available | Rejected | Too weak evidence |
| Venous thrombosis and pulmonary embolism (2016) | Avoid systemic thrombolytic therapy in patients with pulmonary embolism and a moderate risk of death, since it does not decrease overall mortality but does increase the risk of serious bleeding. | B | Accepted |  |
|  | Avoid routine systemic thrombolytic therapy in patients with deep venous thrombosis. | B | Accepted |  |
| Wet age-related macular degeneration (2016) | Avoid ranibizumab and aflibercept as first line treatments for age related macular degeneration due to high costs. | B | Accepted |  |
|  | Do not start VEGF suppressor treatment in patients with age related macular degeneration, if it is not expected to increase their functional capacity or quality of life, or if the harms and risks of the treatment are estimated to exceed the benefits. | No evidence summary available | Accepted |  |
|  | Avoid dosing bevasizumab and ranibizumad every 4-6 weeks in treating wet age-related macular degeneration. | C | Rejected | Complex issue |
|  | Do not continue VEGF suppressor treatment, if it is found to be ineffective, or the harms and risks are considered to exceed the benefits. | No evidence summary available | Rejected | Complex issue |
| Diagnosis of cardiac infarction |  |  | No suggestions |  |
| Epileptic seizure (prolonged) |  |  | No suggestions |  |
| Insulin-deficient diabetes |  |  | No suggestions |  |
| Schizophrenia |  |  | No suggestions |  |

## References

[1]=Bagley LJ. Imaging of neurological emergencies: trauma, hemorrhage, and infarction. Semin Roentgenol. 1999;34:144-59.

[2]=Fiser SM, Johnson SB, Fortune JB. Resource utilization in traumatic brain injury: the role of magnetic resonance imaging. Am Surg. 1998;64:1088-9.

[3]=Gentry LR, Godersky JC, Thompson, Dunn VD. Prospective comparative study of intermediate-field MR and CT in the evaluation of closed head trauma. AJR Am J Roentgenol. 1988;150:673-82.

[4]=Kelly AB, Zimmerman RD, Snow RB ym. Head trauma: comparison of MR and CT--experience in 100 patients. AJNR Am J Neuroradiol. 1988;9:699-708.

[5]=Ashwal S, Holshouser BA. New neuroimaging techniques and their potential role in patients with acute brain injury. J Head Trauma Rehabil. 1997;12:13-35.

[6]=Paterakis K, Karantanas AH, Komnos A, Volikas Z. Outcome of patients with diffuse axonal injury: the significance and prognostic value of MRI in the acute phase. J Trauma. 2000;49:1071-5.

[7]=Firsching R, Woischneck D, Diedrich M, Klein S, Rückert A, Wittig H, et al. Early magnetic resonance imaging of brainstem lesions after severe head injury. J Neurosurg 1998;89:707-12

[8]=SEARCH Collaborative Group, Link E, Parish S, Armitage J, Bowman L, Heath S, et al. SLCO1B1 variants and statin-induced myopathy--a genomewide study. N Engl J Med. 2008;359:789-99.

[9]=Egan A, Colman E. Weighing the benefits of high-dose simvastatin against the risk of myopathy. N Engl J Med. 2011;365:285-7.

[10]=FDA Drug Safety Communication: New restrictions, contraindications, and dose limitations for Zocor (simvastatin) to reduce the risk of muscle injury. <https://www.fda.gov/drugs/drug-safety-and-availability/fda-drug-safety-communication-new-restrictions-contraindications-and-dose-limitations-zocor> (2017). Accessed 4 January 2018.

[11]=Antico A, Pagani M, Vescovi PP, Bonadonna P, Senna G. Food-specific IgG4 lack diagnostic value in adult patients with chronic urticaria and other suspected allergy skin symptoms. Int Arch Allergy Immunol. 2011;155:52-6.

[12]=Stapel SO1, Asero R, Ballmer-Weber BK, Knol EF, Strobel S, Vieths S, et al. Testing for IgG4 against foods is not recommended as a diagnostic tool: EAACI Task Force Report. Allergy. 2008;63:793-6.

[13]=Jensen MD, Ryan DH, Apovian CM, Ard JD, Comuzzie AG, Donato KA, et al. 2013 AHA/ACC/TOS guideline for the management of overweight and obesity in adults: a report of the American College of Cardiology/American Heart Association Task Force on Practice Guidelines and The Obesity Society. Circulation. 2014;129 Suppl:102-38

[14]=Coxib and traditional NSAID Trialists' (CNT) Collaboration, Bhala N, Emberson J, Merhi A, Abramson S, Arber N, et al. Vascular and upper gastrointestinal effects of non-steroidal anti-inflammatory drugs: meta-analyses of individual participant data from randomised trials. Lancet. 2013;382:769-79.

[15]=Burnett AE, Mahan CE, Vazquez SR, Oertel LB, Garcia DA, Ansell J. Guidance for the practical management of the direct oral anticoagulants (DOACs) in VTE treatment. J Thromb Thrombolysis. 2016;41:206-32.

[16]=Beakley BD, Kaye AM, Kaye AD. Tramadol, Pharmacology, Side Effects, and Serotonin Syndrome: A Review. Pain Physician. 2015;18:395-400.

[17]=Abadie D, Rousseau V, Logerot S, Cottin J, Montastruc JL, Montastruc F. Serotonin Syndrome: Analysis of Cases Registered in the French Pharmacovigilance Database. J Clin Psychopharmacol. 2015;35:382-8.

[18]=Perk J, De Backer G, Gohlke H, Graham I, Reiner Z, Verschuren M, et al.. European Guidelines on cardiovascular disease prevention in clinical practice (version 2012). The Fifth Joint Task Force of the European Society of Cardiology and Other Societies on Cardiovascular Disease Prevention in Clinical Practice (constituted by representatives of nine societies and by invited experts). Eur Heart J. 2012;33:1635-701.

[19]=Gibbons RJ, Abrams J, Chatterjee K, Daley J, Deedwania PC, Douglas JS, et al. ACC/AHA 2002 guideline update for the management of patients with chronic stable angina--summary article: a report of the American College of Cardiology/American Heart Association Task Force on Practice Guidelines (Committee on the Management of Patients With Chronic Stable Angina). Circulation. 2003;107:149-58.
